# Few, Activity-Dependent, and Ubiquitous VGLUT1/VGAT Terminals in Rat and Mouse Brain

**DOI:** 10.3389/fncel.2017.00229

**Published:** 2017-08-08

**Authors:** Giorgia Fattorini, Chiara Ciriachi, Fiorenzo Conti

**Affiliations:** ^1^Department of Experimental and Clinical Medicine, Section of Neuroscience and Cell Biology, Università Politecnica delle Marche Ancona, Italy; ^2^Center for Neurobiology of Aging, Istituto Nazionale di Riposo e Cura per Anziani – Istituto di Ricovero e Cura a Carattere Scientifico Ancona, Italy; ^3^Fondazione di Medicina Molecolare, Università Politecnica delle Marche Ancona, Italy

**Keywords:** VGLUT1, VGAT, co-localization, E/I balance

## Abstract

In the neocortex of adult rats VGLUT1 and VGAT co-localize in axon terminals which form both symmetric and asymmetric synapses. They are expressed in the same synaptic vesicles which participate in the exo-endocytotic cycle. Virtually nothing, however, is known on whether VGLUT1/VGAT co-localization occurs in other brain regions. We therefore mapped the distribution of terminals co-expressing VGLUT1/VGAT in the striatum, hippocampus, thalamus, and cerebellar and cerebral cortices of rats and mice. Confocal microscopy analysis revealed that, in both rat and mouse brain, VGLUT1/VGAT+ terminals were present in all brain regions studied, and that their percentage was low and comparable in both species. These results provide the first demonstration that co-expression of VGLUT1 and VGAT is a widespread phenomenon. Since VGLUT1/VGAT+ axon terminals are regulated in an activity-dependent manner and co-release glutamate and GABA, we hypothesize that, though not numerous, they can contribute to regulating excitation/inhibition balance in physiological conditions, thereby playing a role in several neurological and psychiatric diseases.

## Introduction

Glutamate and γ-aminobutyric acid (GABA) are the most important excitatory and inhibitory neurotransmitters in central nervous system, respectively (Conti and Weinberg, 1999; Cherubini and Conti, 2001). Replenishment of glutamatergic and GABAergic synaptic vesicles, a fundamental step in synaptic physiology, is mediated by specific vesicular transporters termed VGLUT1-3 (Gras et al., 2002; Fremeau et al., 2004; Takamori, 2006) and VGAT, respectively (McIntire et al., 1997; Sagne et al., 1997; Takamori et al., 2000).

Safiulina et al. (2006) reported that VGLUT1 and VGAT co-localize in developing hippocampal mossy fibers terminals. Subsequently, we demonstrated in rat adult neocortex that VGLUT1 and VGAT are co-localized in a subset of axon terminals which form both symmetric and asymmetric synapses, that they are sorted to the same vesicles, and that these vesicles participate in the exo-endocytotic cycle (Fattorini et al., 2009). More recently, we showed that glutamatergic and GABAergic responses can be recorded from rat cortical neurons in cultures, indicating the occurrence of glutamate and GABA co-release from neurons co-expressing VGLUT1 and VGAT (mixed synapses), and that the percentage of mixed synapses is regulated in an activity-dependent manner (Fattorini et al., 2015).

To date, very little is known on the occurrence of VGLUT1/VGAT co-localization in other brain regions, (see below). Based on their activity-dependent regulation, terminals co-expressing VGLUT1/VGAT appear suitable to control excitation–inhibition (E/I) balance; and on this basis we hypothesize that they may exhibit a widespread distribution in the brain. In order to test this hypothesis, we investigated the occurence of VGLUT1/VGAT terminals in different brain regions.

## Co-Expression of VGLUT1 and VGAT is A Widespread Phenomenon

**FIGURE 1:**
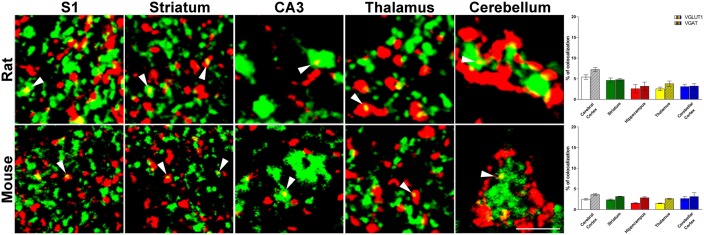
Confocal analysis of rat and mouse brain VGLUT1(green)/VGAT+(red) terminals. Cerebral cortex: layers II-III of S1; Striatum: caudate-putamen (CPu) nucleus; Hippocampus: Stratum Lucidum of CA3; Thalamus: anterodorsal nucleus (AD); Cerebellar cortex: granular layers. Arrowheads point to example of co-localization between VGLUT1 and VGAT. After perfusion brains were removed stereotaxically in order to obtain the regions of interest (Paxinos and Watson, 1986; Paxinos and Franklin, 2001): –0.3 to –6.8 mm from the bregma for rat brain; 0.0 to –3.6 mm from the bregma for mouse brain. Sections were incubated for 1 h in newborn calf serum (NBCS; 10% in PB with 0.2% Triton X-100), and then for 2 h at room temperature plus overnight at 4°C in a solution containing a mixture of anti-VGAT (made in rabbit; Synaptic System, Germany, #131003, RRID:AB_887869; 1:500 rat, 1:400 mouse) (Takamori et al., 2000) and, only in rat experiments, anti-VGLUT1 (made in guinea-pig; Millipore, Billerica, MA, United States, AB5905, RRID:AB_2301751; 1:1000) (Melone et al., 2005) primary antibodies, for mouse experiments we use fluorescent VGLUT1 (Venus) knock-in mouse (Herzog et al., 2011). The next day, sections were incubated in a mixture of CY3 (Jackson ImmunoResearch, West Grove, PA, United States, #711-166-152; 1:250) and (only in rat experiments) Alexa-488 (Jackson Immunoresearch, West Grove, PA, United States, #706-546-148; 1:250). Images were acquired using a Leica TCS-SP2 confocal laser microscope equipped with an argon (488 nm) and a helium/neon (543 nm) using a × 63 oil immersion lens (numerical aperture 1.4; pinhole 1.0 and image size 512 × 512 pixels, yielding a pixel size of 0.155 μm (rat) and 0.133 μm (mouse) from a plane in which the resolution of both stains was optimal and never > 1.8 μm from the surface (Melone et al., 2005). Green and red channels were acquired sequentially. To improve the signal/noise ratio, 10 frames/image were averaged. Images were deconvolved using Iterative Deconvolve 3D plugin (Dougherty, 2005) of ImageJ software (v. 1.48; NIH). Scale bars: 5 μm. Quantitative analysis of VGLUT1/VGAT co-localization in rat (up) and mouse (down) brain regions. The results show that the percentage of VGLUT1/VGAT+ terminals is comparable across regions and species. Three–four animals/regions. Data represent mean ± S.E.M.

Confocal microscopy analysis revealed that in both rat and mouse brain VGLUT1/VGAT+ terminals were present in comparable amount in all brain regions studied (**Figure 1** and **Table 1**).

**Table 1:** VGLUT1-VGAT colocalization in rat and mouse brain subregions.

	RAT		MOUSE	
	% of VGLUT1	% of VGAT	% of VGLUT1	% of VGAT
**Cerebral Cortex**				
S1	6.7 ± 0.7	8.4 ± 0.4	2.4 ± 0.4	2.8 ± 0.4
M1	4.2 ± 0.5	6.1 ± 0.7	2.6 ± 0.2	4.4 ± 0.7
**Striatum**				
* Caudate-Putamen*	4.6 ± 0.6	4.8 ± 0.3	2.4 ± 0.2	3.1 ± 0.1
**Hippocampus**				
**Dentate Gyrus**				
* Molecular layer*	3.7 ± 1.7	4.5 ± 1.6	2.1 ± 0.2	3.3 ± 0.1
* Polymorphic layer*	2.6 ± 1.0	2.7 ± 1.1	0.9 ± 0.2	1.1 ± 0.2
**CA3**				
* Stratum oriens*	3.2 ± 1.4	3.8 ± 1.3	2.2 ± 0.2	4.3 ± 0.3
* Stratum lucidum*	3.1 ± 0.8	3.7 ± 0.7	1.6 ± 0.4	2.2 ± 0.3
* Stratum radiatum*	2.1 ± 0.6	2.0 ± 0.1	1.5 ± 0.2	2.6 ± 0.2
**CA1**				
* Lacumosum-moleculare*	1.7 ± 0.9	2.9 ± 1.3	1.3 ± 0.1	3.8 ± 0.5
* Stratum radiatum*	1.7 ± 0.8	2.6 ± 0.9	1.3 ± 0.1	2.6 ± 0.5
**Thalamus**				
AD	4.5 ± 0.6	7.1 ± 1.3	2.0 ± 0.3	3.6 ± 0.6
AV	2.7 ± 0.7	4.9 ± 1.2	1.7 ± 0.2	3.6 ± 0.5
PVA	3.7 ± 0.7	4.6 ± 0.7	2.2 ± 0.2	2.8 ± 0.4
PT	2.3 ± 1.0	3.5 ± 1.2	1.0 ± 0.3	2.2 ± 0.3
IAM	3.0 ± 1.0	4.5 ± 0.9	1.3 ± 0.3	2.2 ± 0.2
Rh	3.8 ± 0.5	3.8 ± 0.5	1.7 ± 0.4	2.3 ± 0.2
Re	3.4 ± 0.5	3.8 ± 0.5	2.1 ± 0.3	2.5 ± 0.2
CM	2.9 ± 0.1	4.2 ± 0.4	1.1 ± 0.3	1.8 ± 0.4
CL	2.2 ± 0.6	3.1 ± 0.6	1.3 ± 0.3	1.9 ± 0.2
PF	2.6 ± 0.4	2.4 ± 0.5	1.5 ± 0.4	1.1 ± 0.4
MD	1.2 ± 0.6	2.5 ± 0.9	1.2 ± 0.2	2.4 ± 0.4
VL	2.8 ± 0.9	4.1 ± 0.9	1.5 ± 0.2	3.1 ± 0.4
VPL	1.2 ± 0.4	3.1 ± 1.0	1.5 ± 0.6	2.8 ± 0.5
VPM	1.6 ± 0.6	3.7 ± 1.0	1.0 ± 0.2	2.5 ± 0.3
DLG	1.5 ± 0.5	2.7 ± 1.0	1.4 ± 0.4	2.9 ± 0.7
MGV	1.3 ± 0.2	3.0 ± 0.5	1.3 ± 0.5	3.2 ± 0.7
**Cerebellar Cortex**				
* Molecular layer*	1.7 ± 0.2	3.3 ± 0.6	1.4 ± 0.4	3.1 ± 0.7
* Granular layer*	6.5 ± 1.1	3.0 ± 0.7	4.8 ± 1.5	3.4 ± 1.4


*Cerebral cortex: layers II-III of S1 and M1 (six fields/section; rat: one section/animal, four rats; mouse: two sections/animal, three mice); Striatum: caudate-putamen (CPu) nucleus (six fields/section, two section/animal, three animals); Hippocampus: Molecular layer and Polymorphic cell layer of the Dentate Gyrus; Stratum Oriens, Stratum Lucidum and Stratum Radiatum of CA3; Stratum Lacunosum-Moleculare and Stratum Radiatum of CA1 (three fields/layer/section, two sections/animal, three animals); Thalamus: anterior nuclear group [anterodorsal nucleus (AD), anteroventral nucleus (AV)]; dorsomedial nucleus (MD); midline nuclear group [paratenial nucleus (PT), paraventricular nucleus (PVA), interanteromedial nucleus (IAM), reuniens nucleus (Re), rhomboidal nucleus (Rh)]; intralaminar nuclei [central lateral nucleus (CL), central medial nucleus (CM), parafascicular nucleus (PF)]; ventral nuclear group [ventral lateral nucleus (VL), ventral posterolateral (VPL), ventral posteromedial (VPM)]; medial geniculate complex [medial geniculate nucleus (MGV)]; dorsal lateral geniculate nucleus (DLG; three fields/nucleus/section, two sections/animal, three animals); Cerebellum: Molecular and granular layers of cortex (six fields/layer/section, two sections/animal, three animals). Data represent mean ± S.E.M*.

In rat brain, the percentage of VGLUT1+ puncta co-expressing VGAT was 5.4 ± 0.5% in cerebral cortex; 4.6 ± 0.6% in striatum; 2.6 ± 1.0% in hippocampus; 2.5 ± 0.4% in thalamus; and 3.1 ± 0.6% in cerebellar cortex, while the percentage of VGAT+ puncta co-expressing VGLUT1 was 6.0 ± 0.5% in cerebral cortex; 4.8 ± 0.3% in striatum; 3.2 ± 1.0% in hippocampus; 3.8 ± 0.7% in thalamus; and 3.2 ± 0.6% in cerebellar cortex (**Figure 1**). Details on individual nuclei, layers or sub-regions are presented in **Table 1**.

Mice are more widely used than rats for generating transgenic animals, an approach that could be useful in the future to define the functional role of the system of VGLUT1/VGAT co-expressing terminals; for this reason, we performed the same analysis in this species. In mouse brain, the percentage of VGLUT1+ puncta co-expressing VGAT was 2.5 ± 0.2% in cerebral cortex; 2.4 ± 0.2% in striatum; 1.6 ± 0.1% in hippocampus; 1.5 ± 0.07% in thalamus; and 2.7 ± 0.5 in cerebellar cortex, while the percentage of VGAT+ puncta co-expressing VGLUT1 was 3.6 ± 0.2% in cerebral cortex; 3.1 ± 0.07% in striatum; 2.8 ± 0.2 in hippocampus; 2.6 ± 0.1 in thalamus; and 3.1 ± 0.9 in cerebellar cortex (**Figure 1**). Details on individual nuclei, layers or sub-regions are given in **Table 1**.

## VGLUT1/VGAT Terminals may Contribute to E/I Balance in A Multitude of Brain Circuits

In the last few years, several papers have documented that VGLUT1 and VGAT are co-expressed in cerebral cortex, hippocampus and cerebellum of rats and mice (Gutierrez, 2005; Safiulina et al., 2006; Uchigashima et al., 2007; Fattorini et al., 2009; Zander et al., 2010; Beltran and Gutierrez, 2012). To date, the occurrence of VGLUT1/VGAT co-expression in other brain regions of both species is poorly understood. Here, we showed for the first time that co-expression of VGLUT1 and VGAT is a widespread phenomenon, and that the co-localization of the two vesicular transporters is not markedly different between the two species, an observation that might be of some help for studying the role of VGLUT1/VGAT co-expression in animal models of neurological and psychiatric diseases.

The main data emerging from this study is that VGLUT1/VGAT+ terminals are present in all the anatomical structures studied of both species. The percentage of VGLUT1/VGAT+ terminals is regulated in an activity-dependent manner: reducing the activity of the neuronal network by addition of glutamate receptor antagonists to the cultures decreases the percentage of mixed synapses, whereas reducing spontaneous inhibition with bicuculline increases them (Fattorini et al., 2015). These findings support the idea that activity-dependent regulation of glutamate and GABA co-release might play a role in regulating E/I balance in cortical microcircuits, thereby contributing to regulate brain function in normal and pathological conditions. The present demonstration that VGLUT1/VGAT co-expression occurs in all brain regions studied suggests that these terminals may contribute to adjust E/I balance in a multitude of brain circuits. For example, it is well known that an abnormal increase of glutamatergic and/or an abnormal decrease of GABAergic transmission in hippocampus promotes neuronal hyperexcitability and hypersynchronization, and may sustain epileptic networks (e.g., Bonansco and Fuenzalida, 2016). The present evidence of a population of hippocampal VGLUT1/VGAT+ axon terminals, demonstrated here for the first time in adult normal animals, raises the possibility that their dysregulation may generate E/I imbalance. In addition, controlling inhibitory and excitatory sources (both local and extrinsic) in higher-order thalamic nuclei may contribute to thalamic oscillations (Fogerson and Huguenard, 2016), implying that VGLUT1/VGAT+ terminals-mediated E/I imbalance in these circuits may play a crucial role in some forms of epilepsy. Finally, the network of parvalbumin+ fast-spiking interneurons has attracted much interest in autism spectrum disorders (ASDs), and parvalbumin knockout mice exhibit behavioral phenotypes resembling core symptoms of the diseases. In the striatum of these mice, E/I balance is altered by modification of both inhibitory and excitatory synaptic transmission, leading to the hypothesis that down-regulation of parvalbumin might be central to the neurobiological basis of the diseases (Wohr et al., 2015). In this context, it is worth noting that in fast-spiking-enriched cultures the percentage of VGLUT1/VGAT+ terminals is increased compared to controls (Fattorini et al., 2015), thus making it conceivable that VGLUT1/VGAT+ terminals in striatum (as well as in other brain regions) may play a role.

## Conclusion

We showed that VGLUT1/VGAT+ axon terminals are present in all structures studied in both rat and mouse brain. Their percentage is relatively low, but given their activity-dependent regulation, it is conceivable that they may play a role in both physiological regulation of E/I and in the pathophysiology of several neurological and psychiatric diseases.

## Ethics Statement

All experimental procedures involving animals and their care were carried out in accordance with National laws and policies (D.L.26, March 14, 2014), and with the European Community Council Directive guidelines (2010/63/UE); all procedures were approved by the local authority veterinary services (Università Politecnica delle Marche).

## Author Contributions

GF developed the concept, analyzed the data, and wrote the manuscript. CC performed the experiments and analyzed the data. FC developed the concept and wrote the manuscript.

## Conflict of Interest Statement

The authors declare that the research was conducted in the absence of any commercial or financial relationships that could be construed as a potential conflict of interest.
